# Camphorated Oil (*Lin. Camphoræ, U.S.*) and Cotton, as a Local Application in Erysipelas

**Published:** 1866-12

**Authors:** B. H. Cheney

**Affiliations:** Joliet, Ill.


ARTICLE XLVII.

### CAMPHORATED OIL (*Lin. Camphoræ, U.S.*) AND COTTON, AS A LOCAL APPLICATION IN ERYSIPELAS.

By B. H. CHENEY, M.D., Joliet, Ill.

Having, for some years past, been in the habit of using in erysipelas a local application at once simple, agreeable to the patient, and comparing favorably in my experience with any other, I venture to submit it to those of the profession who may not already have given it a trial. This is the *linimentum camphoræ* of the U.S. Dispensatory, commonly known as camphorated oil.

I should say, at the outset, that I by no means claim originality in the use of this application in the disease mentioned, for it is very probable that others may be in the habit of using the same. But in no medical work, to which I have access, can I find it alluded to, in the long catalogues of local applications in the treatment of erysipelas, nor am I aware that attention has ever been called to it through the medium of medical journals.

Although subscribing to the doctrine of WATSON, that erysipelas is an exanthematous fever, characterized by its peculiar eruption upon the face and head, and that similar affections of the skin simply, occurring elsewhere, are more properly erysipelatous inflammations, yet this is no reason for omitting all local treatment whatever, nor even for judging that local remedies are useless.

My practice in the use of this remedy is to saturate with the oil one side of a thin sheet of cotton-batting, of the required shape and size, and apply it to the affected part. I so cover all that portion of the skin invaded by the disease, and allow the cotton to extend to some distance beyond, over the sound skin. The whole of the face is frequently thus covered, the apertures being, of course, left for the mouth and nostrils. The cotton should be changed morning and night; and it is well to throw the pieces which are removed at once into the fire, as one is then on the safe side of the question of contagion or the communicability of the disease from such a source. I am in the habit of having the fresh pieces of cotton all ready, and of applying them immediately as the others are removed, though I do not contend that this precaution is essential. The general or constitutional treatment is the usual one of diaphoretics and tonics, especially the tinct. ferri muriat., with such other remedies as may be called for by existing indications.

The general tendency of opinion among the leading minds in the profession, is, probably, to the belief that the question of local applications in this disease is, to a great extent, an indifferent one. But, even in this case, the camphorated oil has much, negatively, to recommend it over other and more disagreeable remedies. I have known individuals, whose sufferings from the repeated application of the tincture of iodine were great, the skin being irritated and smarting constantly. I am not prepared to say that the final result would have been materially altered by the use of the camphorated oil in these cases, but I am convinced that the iodine is in no way superior to the latter remedy in efficacy for the treatment of this disease, and the suffering was, therefore, wholly unnecessary.

There are those who fear to leave the beaten track, not so much from blind adherence to established usage, as from a commendable conscientiousness, lest, in so doing, they trifle with human life. For the satisfaction of such, who may be loth to trust a bad case without the tinct. of iodine or solution of nitrate of silver to arrest the progress of the disease, I will say that I have tested the use of the camphorated oil in cases of all grades and characters, from the mildest manifestation of the disease, to cases truly malignant, in military hospitals, and in private practice. The results under this treatment were remarkably favorable, compared with cases under other forms of treatment, or with any statistics, so far as I know.

Those, on the other hand, who regard the treatment of erysipelas as of some importance, and those who agree with EBERLE, that "there exists a close analogy between the inflammation of erysipelas and that produced by a scald or slight burn," will observe that all the consequent indications are answered by the camphorated oil. The oil is soothing, the camphor a gentle stimulant to the inflamed surface, and the cotton effectually excludes air. To the latter power, that of excluding air, is probably due much of the efficacy of all local applications in this disease.

In cases of erysipelatous inflammation locally manifested elsewhere than the face and head, and especially where traumatic, the use of iodine, or some remedy more positive in its effects than the oil, seems to be called for. Whether there be the same difference existing between erysipelas proper and an erysipelatous inflammation, as is found, according to VIRCHOW,* between the latter and the ordinary exanthemata, or what lines of pathological distinction may be drawn between them, it is not my purpose or place here to inquire. But from the greater tendency in the local disease to suppuration in the subcutaneous areolar tissue, to gangrene, etc., it is evident that there is a strong local manifestation of the blood dyscrasy, and as the disease has no definite time to run, its course is more uncertain and calls for more vigorous local measures.

On the other hand, no local application whatever will stay the progress of erysipelas proper until the fever has run its course, any more than they will stay the progress of measles, scarlatina, or small-pox.

### ARTICLE XLVIII.

### A FEW PRACTICAL SUGGESTIONS ON THE MANAGEMENT OF CHRONIC ULCERS.

BY R. DEXTER, M.D., of Chicago.

If an ulcer has become chronic, it is owing to a cause or causes that tend to subvert the reparative powers of the system, thus thwarting nature's efforts to heal the breach. These causes

* Virchow, Cell. Path., L. viii., p. 200.

